# Overview of Methods for Overcoming Hindrance to Drug Delivery to Tumors, with Special Attention to Tumor Interstitial Fluid

**DOI:** 10.3389/fonc.2015.00165

**Published:** 2015-07-23

**Authors:** Gianfranco Baronzio, Gurdev Parmar, Miriam Baronzio

**Affiliations:** ^1^Integrative Oncology Section, Medical Center Kines, Milan, Italy; ^2^Integrated Health Clinic, Fort Langley, BC, Canada

**Keywords:** tumor interstitial fluid, tumor interstitial fluid pressure, drug delivery systems, chemotherapy, adjuvant, vascular normalization

## Abstract

Every drug used to treat cancer (chemotherapeutics, immunological, monoclonal antibodies, nanoparticles, radionuclides) must reach the targeted cells through the tumor environment at adequate concentrations, in order to exert their cell-killing effects. For any of these agents to reach the goal cells, they must overcome a number of impediments created by the tumor microenvironment (TME), beginning with tumor interstitial fluid pressure (TIFP), and a multifactorial increase in composition of the extracellular matrix (ECM). A primary modifier of TME is hypoxia, which increases the production of growth factors, such as vascular endothelial growth factor and platelet-derived growth factor. These growth factors released by both tumor cells and bone marrow recruited myeloid cells form abnormal vasculature characterized by vessels that are tortuous and more permeable. Increased leakiness combined with increased inflammatory byproducts accumulates fluid within the tumor mass (tumor interstitial fluid), ultimately creating an increased pressure (TIFP). Fibroblasts are also up-regulated by the TME, and deposit fibers that further augment the density of the ECM, thus, further worsening the TIFP. Increased TIFP with the ECM are the major obstacles to adequate drug delivery. By decreasing TIFP and ECM density, we can expect an associated rise in drug concentration within the tumor itself. In this overview, we will describe all the methods (drugs, nutraceuticals, and physical methods of treatment) able to lower TIFP and to modify ECM used for increasing drug concentration within the tumor tissue.

## Introduction

To produce its effects, a drug should reach the target tissue in a uniform and selective way. Although this effect has not been accomplished for any disease, with any drug currently used, this is true more than ever for tumors. Chemotherapy alone has not proven its effectiveness and efficacy; in fact, studies over 5 years have produced a paltry percentage of 2.1% (1).

Although several criticisms can be moved to this article, it is the only one that has produced reliable data on a large population and has shown which tumors may benefit from the use of chemotherapy. The work does not analyze the reasons for this failure. In fact, it was not possible to analyze the mode of drug administration, the various cocktails used, and if they were properly prepared. Furthermore, it was not easy to study all the anatomical parameters (currently known by *in vitro* and animal studies) that were able to decrease the arrival of the drugs to the tumor. The first studies that have taken into account the reasons why chemotherapeutics are not able to achieve their antitumor effect are to ascribe to Jain et al. (2). These authors studied the pharmacokinetics of methotrexate (MTX) in two transplanted-animal carcinoma: Walker 256carcinoma (W256) and hepatoma 5123 (H5123). A difference was present in the two tumors regarding the distribution of MTX. The uptake of drugs by H5123 was conditioned by the plasmatic concentration, whereas, in the W256, the tissue barriers conditioned it. It is interesting to report the methods used by these authors. The authors (2) studied the pharmacokinetics of MTX in W256 and H5123 by transplanting the tumors in three different ways. The first method of transplantation was the standard implantation of tumor frustules in the subcutaneous tissue. The second method was the implantation of a Millipore chamber inside the tumor mass for sampling tumor interstitial fluid (TIF) (3). The third method used tumor implantation to obtain a tumor supply by the host connecting it to a single artery and vein (4, 5). The single artery and vein connection is a superb method for studying tumor blood perfusion and vasoactive drug effects, metabolites, and drug characterization (6). To determine experimentally the release of drugs into tumors, Jain and coworkers (7–10) used several methods of study. One method was the isolated organ of Gullino, as previously reported. The other methods were the preparations of microcirculatory units. One method was the “Window technique” (7), and the other was a new angiogenesis assay (9, 10). This new assay was able to quantify angiogenesis, red blood cell velocity, microvascular permeability, pH, and growth factors (9, 10). From these early pharmacokinetic studies and the combined use of these experimental methods, Jain concluded that the drugs do not come easily to the tumor mass (7). Jain also stressed that different barriers prevent their arrival and that increased interstitial pressure is the main impediment (11). Other researchers have confirmed the existence of these anatomical and physiological barriers (12, 13). As recently pointed out by Monsky (12), barriers related to the anatomy and physiology of the tumor are the tumor vasculature, the interstitial space, and the same tumor cells.

Associated with increased interstitial pressure, the irregular vasculature is responsible for the decreased intake of drugs (11, 12, 14, 15).

## Tumor Vasculature Interstitial Fluid Formation, Increase of Interstitial Fluid Pressure

As long as the tumor in its growth does not exceed a distance from the nourishing vessels >1–2 mm^3^, the tumor remains well oxygenated and nourished. Once this volume is exceeded, many cells become hypoxic and undernourished (15). At this point, a mechanism common to many hypoxic situations is triggered that seeks to bring nourishment and oxygen to these suffering cells (15, 16). The defense mechanism triggered by a transcription factor called hypoxia-inducible factor (HIF), regulate the production of several growth factors and trigger angiogenesis (17). Among growth factors, vascular endothelial growth factor A (VEGF-A) and platelet-derived growth factor (PDGF) are the most studied (18–22). VEGF and PDGF not only exert mitogenic effects on endothelial cells (21, 23, 24) but also sustain inflammatory reactions. In fact, VEGF and PDGF recruit myeloid and immature cells from the blood marrow. These cells contribute to building the new vasculature (25–28).

As reported by Narang (14), the excessive quantity of vascular cytokines and growth factors in the tumor microenvironment (TME) determines an irregular growth of vessels compared to its normal counterparts. In conclusion, the tumor vasculature is a defective vasculature compared to that appearing during wound repair or in normal tissues (19, 23). In fact, tumor vasculature appears with a non-ordered 3D branching, lacking smooth muscles, and pericytes, with a scarce or missing innervation with irregular basement membrane (14, 27). The architecture of the tumor vasculature is aberrant (27) and develops in similar way in many types of tumors (29, 30). Furthermore, Azzi and Nagy (31, 32) describe a loss of cellular junctions integrity. This loss of integrity is responsible for the increased permeability of the tumor neovessels (31, 32). Another factor contributing to increasing the permeability of tumor vasculature is the excessive production of VEGF. In fact, a study by Roberts and Palade (33) has demonstrated that VEGF increases the permeability of postcapillary venules, increasing their fenestration. This fenestration effect was also present in the endothelium not usually fenestrated, such as skin and muscle. Weis and Cheresh (34) reported a similar increase in permeability and edema in cancer and in ischemic tissues. These structural effects are the result of biochemical defects induced by VEGF (35). The simultaneous presence of increased permeability, lack of lymphatic drainage (36, 37), and chronic inflammation in the TME carries to an accumulation of fluid in the interstitium (38, 39). The chronic inflammatory reaction is elicited by VEGF (40) and other cytokines/chemokines present in the TME (41, 42). Various authors demonstrated the increased interstitial fluid accumulation (3, 38, 43–45). To understanding better this fluid accumulation, a digression on the forces that govern the exchange of liquids in capillaries is useful. Ernest Starling (46) formulated the various factors that regulate the filtration of liquids through the vascular wall and the exchange of fluids between interstitium and plasma in 1896. It is because two major gradient forces present at the level of the capillaries control this transfer the hydrostatic pressure that favors the filtration, and the osmotic pressure gradient, that favors resorption. Mathematically, is expressed as:

(1) Jv=(LpS)[(Pc−Pi)−σ(πc−πi)]

where Jv is the volume flux of fluid (ml/min); Lp is hydraulic conductivity (cm min^−1^ mmHg^−1^); s is the capillary surface area (cm^2^); Pc and Pi are capillary and interstitial fluid hydrostatic pressures, respectively (mmHg); πc and πi are capillary and interstitial colloid (oncotic) pressures, respectively (mmHg); and σ is the osmotic reflection coefficient of the vessel wall (σ 0 if the membrane is fully permeable to transport molecular species and σ 1 if the membrane is impermeable) (38, 46–47). This equation still cannot fully explain the formation of interstitial fluid in pathological situations to complete it, it is necessary to introduce a new parameter that is the flow of liquid removed from the interstitium by the lymphatic system. The equation is modified taking into account the amount of liquid removed by the lymphatics: JL (38, 46, 47, 48):

(2) Jv=(LpS)[(Pc−Pi)−σ(πc−πi)]−JL

In tumor mass, however, these forces are not regulated for various reasons such as capillary tumor pressure The change in hydrostatic pressure along the length of the capillary tumor is lower than that of the normal capillary (MVP). This pressure decrease gives rise, as described by some authors, to a stagnant tumor blood flow (49). Furthermore, the tumor blood viscosity is increased, and this aggravates further the perfusion (50). A second factor is related to the osmotic pressure, slightly elevated in the tumor interstitium, compared to plasma. Another important factor is the composition of the interstitial fluid itself, richer in collagen and glycosaminoglycans that acts almost like a sponge sucking up the interstitial fluid. The last, but probably the most important, factor is that the tumor inside its mass lacks a functioning lymphatic system (36, 37).

The net result of this non-equilibrium is an accumulation of the liquid (TIF) in a confined space, determining an increase in the tumor interstitial fluid pressure (TIFP). The volume occupied by TIF varies between 30 and 60% of tumor water, depending on tumor type, as reported by Gullino (43–45). In addition, other components of TIF vary according to the tumor type studied and to the methodologies used. For example, a difference exists in the protein content using Gullino technique (capsule method) (43) and the method of Sylven and Bois that obtained TIF by micropipettes (51). The same applies to the components of the matrix. For example, glycosaminoglycans have a higher concentration in the TIF compared to normal interstitium (11, 52). Recently, some authors have begun to study the interstitial fluid of patients with cancer proteomics method, in order to obtain new tumor biomarkers (38, 39, 53, 54). Another important aspect is to describe the pressure exerted by the TIF and the consequent interstitial fluid flow (IFF) generated (39). The TIFP following Guyton (55, 56) is physiologically the result of two components: the pressure exerted by the cellular component and that of the gel phase. In the tumor, the tissue pressure (or solid pressure) is made up of growing tumor cells, fibroblasts, and the extracellular matrix (ECM). The gel phase is constituted of the filtrate of tumor vessels in the TIF (57, 58). Before Guyton, average interstitial fluid pressure (IFP) was thought to be near zero or positive, after the use of capsule method it has been found to be near zero or sub-atmospheric (55, 56). Young was the first to measure TIFP and was followed by Gullino and Jain (3, 11, 59). These authors demonstrated in animal and human studies that TIFP is greater than in normal tissue and positive, reaching in particular tumors the value of 100 mmHg (i.e., Melanoma) (range 10–40 mmHg) (60–62). As outlined by Jain, TIFP decreases from the center of the tumor toward the periphery and correlated to volume (60). IFF is the fluid present in the stroma and is poorly drained by lymphatics and maintained by the TIFP gradient (63–65). In fact, as reported by Jain (63), IFF is proportional to the pressure gradient, and its velocity hampers the convective movement of drugs. The velocity of the IFF that affects hydraulic conductivity (K) [cm^2^/mmHg/s] is proportional to the pressure gradient. Its velocity value in tumor tissue ranges from 0.59 to 55 μm/s, which is a higher value compared to normal tissue (0.1–1 μm/s) (64–66). Butler et al. (64) were the first to study the existence of IFF through the micropore chamber method implanted in murine mammary tumors. These authors affirmed that IFF was comparable to lymphatic drainage and takes importance in drug concentration and distribution (64). Several methods have been used to study IIF. Chary and Jain studied the diffusion of albumin in rabbit ear chamber of normal and neoplastic tissue analyzing the fluorescence recovery after photobleaching. These authors founded that the average IFF velocity was about 0.6 μm/s and directed toward the postcapillary venules (67). Munson et al. analyzed the mean velocity found *in vivo* in different cancer types and report a median velocity of 0.5 to 55 μm/s for non-metastatic tumors and values between 10 and 55 for metastatic tumors (65). Other authors followed the development and the flux of IFF by magnetic resonance imaging (MRI) (66, 68). The IFF velocity is dependent on the structure and composition of the extracellular compartment and the physicochemical properties of the drug or solute used (63). The IFF increase, as demonstrated *in vivo* using xeno-engrafted models of various types of human tumors, has its maximum at the periphery of the tumor and its minimum within the tumor mass (66). As outlined by Yao, the content of the interstitium (Collagen fibrils association) and the vascular architecture are the major modifiers of the IFF (69). Other researchers show that IFF is involved with various factors that may enhance tumor progression. In fact, IFF participates in lymphatic dissemination (66, 70) and has an immunomodulatory effect (71). Rutokowski et al. have experimentally demonstrated that IFF increases angiogenesis and lymphangiogenesis. In fact, IFF also acts as a morphoregulator (72) increasing endothelial sprouting, adhesion, permeability, and produces migratory activity in fibroblasts. Furthermore, IFF induces secretion of cytokines and metalloproteinases by these tumor associated fibroblasts (MMPs) (73, 74) (TIF formation is summarized in Box 1).

Box 1 **TIF formation is the result of three important factors simultaneously present in the tumor area.**

They are (a) an unbalanced Starling mechanism acting in tumor microcirculation, (b) increased vascular permeability, due to an abnormal tumor vasculature, (c) a malfunctioned lymphatic system inside the tumor mass. Both these factors generate an increased interstitial pressure (TIFP) and interstitial fluid flow (IFF), moving from the tumor into the near microenvironment. IFF with TIFP hinders drug distribution (11, 38, 39, 63, 72, 211).

## Forces That Govern Drug Distribution from Vascular Wall to Cancer Cells

**Figure 1:**
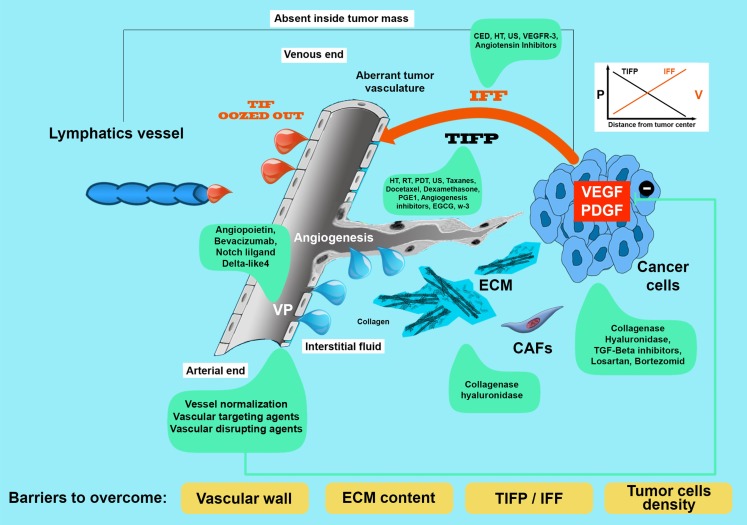
**In this figure, we have tried to illustrate all the barriers (in the yellow rectangles) encountered by a blood-borne drug in its journey from vessel wall to cancer cells**. For every barriers, tumor interstitial fluid pressure (TIFP), IFF, extracellular matrix cell packing, and vascular permeability, we have mentioned in green frames, the methods used to decrease or modulate them. VP, vascular permeability; TIFP, tumor interstitial fluid pressure; IFF, interstitial fluid flow; v, velocity.

The drugs must be divided into drugs with a size ≤1 nm and superior to ≥1 nm. In Table 1, following Multhoff and Vaupel (75), we report the list of drugs with their size. The first obstacle to overcome is the vascular wall (see Figure 1). Transvascular wall mass transport may be diffusive or convective, dependent on osmotic and pressure exiting on the two sides of the vascular wall. Mass transport happens through pores present in the capillary wall. As previous reported tumor vasculature is peculiarly leaky due to an increased number of fenestrae created by VEGF. Disputes regarding the dominant channels that permit the passage of different molecules occurred [see Ref. (76)]. According to Sarin (77), the physiologic upper limit of vascular pore size ranges between 5 and 12 nm. A study by Monsky with the intention of measuring changes in permeability and pore cutoff under increasing dosages of VEGF has demonstrated that it is possible to increase pore size in the range that permits the passage of molecules with ranges between 100 and 300 nm (78). Once the drug has crossed the vascular wall, it must reach the cancer cells traveling along the interstitium. At this point, the interstitial transport of the drug is again dependent on the size. If the size is ≤1 nm (as are the majority of normal chemotherapeutics), travel is governed by diffusion parameters (Fick’s law). Fick’s law is mathematically formulated (11, 79) as:

(3) JD=−(∂C/∂x)

where JD is the diffusive flow of the solute (g/s × m^2^) and D is the diffusion of the solute in the medium (m^2^/s). Even if the molecular size of the molecules are ≤1 nm, they encounter several difficulties in penetrating into tumor mass. These impediments are as follows: (a) the increased distance between the vessel wall and the cancer cells due to the increased volume of tumor interstitium; (b) the diffusion coefficient of tumors; and (c) the presence of a centrifuge flow (IFF) from the tumor center toward the periphery, governed by the IFP. Diffusion coefficient measures are not easy, and Jain (11) describes the methods. The author outlines that D is dependent on many factors, such as water content, molecular weight of the solute, temperature, configuration, and charge and binding of the solute with matrix components. Molecules, such as nanoparticles or monoclonal antibodies, are transported into the interstitium to cancer cells by convective flow that is formulated following Pusenjak and Miklavcic (79) and Jain (11) as

(4) JC=−C×RF×K×(∂p/∂x)

where JC is the convective flow of solute (g/s × m^2^); C is the concentration of solute (g/m^3^); RF is the retardation factor (solute convective velocity/solvent convective velocity); K is the hydraulic conductivity (m^2^/P·a × s); and ∂p/∂x is the hydraulic pressure gradient. Hydraulic conductivity K and the retardation factor seem dependent on the quantity of polysaccharides and the quantity of water present in the interstitium (11, 79, 80). RF is a parameter dependent on the solute (structure, content in water, and molecular properties) (81).

**Table 1:** **Drugs and particles dimension according to the organization for standardization**.

Type of nanoparticles	Size (nm)
Gold nanoparticles	2.5
Monoclonal antibodies	10–15
Oncolytic viruses	30–40
Magnetic nanoparticles	15–100
Liposome encapsulated doxorubicin	80–130
Gadolinium-based nanoparticles	115
Albumin–paclitaxel nanoparticles	130

Modified from Multhoff and Vaupel (75)*.

http://wwwiso.org/iso/home/search.htm?qt=nanoparticles&sort=rel&type=simple&published=on

Another important factor that prevents the arrival of drugs over the interstitial pressure increase but is dependent on it is IFF. Recently, to overcome the complexity of exiting through tumor vascularization, permeability, lymphatics, IFF, and drug transport, Welter et al. have developed a computational model (82). This model predicts interesting behaviors of TIFP and IFF on drug transport in the presence of a heterogeneous tumor vasculature. The authors conclude that IFF is more responsible than TIFP in obstructing drug penetration (82) (Box 2). This effect is, of course, according to this model more at the tumor periphery where IFFs’ velocity is greater. This last effect is in agreement with the observations of Butler et al. (11, 64) that describe that TIF oozes out a quantity of four to five times greater than that in subcutaneous tissue. The model of Welter also predicts that reducing tumor leakiness is not as effective as tumor normalization. Increased tumor permeability may increase drug delivery toward the tumor center where TIFP is greater but IFF velocity is less (82). Other authors using another numerical modeling demonstrated that the irregularities of tumor vasculature influences greatly IFF flux and TIFP (83–85).

Box 2 **Drugs (apart their molecular weight and physical properties) (11, 200) distribute inside tumor mass under the pressures of two principal forces: the TIF and the IFF.**

IFF is generated by TIF pressure difference (82, 200). Furthermore, matrix composition plays an important role in hampering drug penetration (212).

## Techniques for Measuring Tumor Interstitial Fluid Pressure

Tumor interstitial fluid can be measured with different techniques that may be divided according to Wiig (86, 87) into acute or chronic, and into invasive and not invasive.

### Acute invasive methods

The invasive methods can be used in an acute way, such as the technique of Wick catheter, or in a chronic way, as with the implanted perforated capsules (86). Hargens (88) subsequently amended the Wick technique developed by Scholander (89). It consists of a needle filled with a saline solution and its ends a suture thread of dacron. This catheter is then inserted into another catheter (that acts as a guide) and positioned in the skin. The Wick catheter connected to a transducer to record the variation in pressure displaces the guide catheter. A variation of this method is that developed by the group of Aukland called the WIN technique (90). Another acute way is the glass micropipettes used by Wiederhielm in the late 1970 (91). The means of adopting the needles to measure the IFP are, according to Guyton, not adequate (55). In fact, the diameter of these needles is often considerably higher, up to 500 times than the interstitial space to measure. We refer the reader to the review of Wiig (86, 87) for a complete list of methods of measurement of interstitial fluid and the critical aspects of these applications. A summary on the advantages disadvantages and clinical utility of these methods is formulated in Table 2 (Box 3) (92–94).

**Table 2:** **Methods for measuring interstitial pressure (IP) in tumors**.

Type	Method	Tip diameter	Advantage	Disadvantage	Clinical utility
A	Needle	0.5 μm	Simplicity	Tissue destruction and trauma	For superficial visible tumors
A*	Win (Wick-in-needle)	23G needle	Versatile, recorded pressure similar to micropipette	Tissue destruction and trauma	For superficial tumors (i.e., melanoma, breast)
A	Micropipette	2–5 μm	Reduced tissue destruction and trauma	Not possible to measure IP at depth ≥800 μm fragility, immobilization of tissue	Only for superficial visible tumors. Extremely delicate
C	Micropore Chamber	D C 0.8–3 cm	Useful for following biochemical and physiological parameters	Animal preparation not simple peculiarly vascular pedicle – not sensitive to TIFP acute change	No, but sometimes used
NIM	MRN		Useful for following various microenvironmental parameters(i.e., oxygen content, tumor vascularity, tumor perfusion)	Construction of special image platform analysis, possible severe side effects to kidneys	Yes: expensive dedicated structure and staff

A, acute method; C, chronic method; DC, diameter of the capsule; MRN, magnetic resonance imaging; NI, non-invasive methods; A*, standard method according to Wiig.

Box 3 **In Table 2, the two groups of methods are compared.**

The advantages and disadvantages briefly analyzed. In every case, the notation of Pusenjak and Miklavcic (79) is correct. Any method of measurement is imperfect but can be used, provided it is reproducible and easy to use. In the specific case of the TIFP, the wick-in-needle technique seems the most used and reproducible, at least in animal studies and in some superficial human tumors (melanoma, breast, cervix, and head and neck tumors) (61, 79). MRI for humans will be the most applicable method even if expensive.

### Chronic methods

The chronic methods of measurement of IFP are obtained by subcutaneously inserting capsules of polyethylene. They differ in the number of holes on their surface and in the diameter. The pressure measurement is, in one case, done by inserting a catheter inside the capsule, while the other type is directly in contact with the interstitial fluid. The measurement is carried out by connecting the catheter to a pressure transducer (86).

Not all the methods briefly described so far are applicable to humans, except the method of Wick used to measure the TIFP of superficial melanoma or lymphoma nodules (95). Recently, a method to determine TIFP non-invasively has been attempted, unsuccessfully correlating the TIFP with the proton relaxation rate of the magnetic resonance (96). From this failure, Hassid and his group showed that using the proton relaxation time of gadolinium makes it possible to determine the IFP and its spatial distribution (97, 98). Gade and his group, using MRI, have gone further and found that it is possible to quantify the uptake of 5-fluorouracil (5-FU) using a collagenase for degrading the ECM collagen (99). Despite the usefulness and versatility of magnetic resonance, its applicability to the patient is not immediate. As suggested by Hassid, we must take into account gadolinium’s side effects (fibrosis) to the kidneys (98).

Another technique able to monitor non-invasively the pharmacokinetics of a drug is positron emission tomography (PET). An example of PETs use is the pharmacokinetics study *in vivo* of carbon-11 labeled docetaxel ([(11)^C^] docetaxel). Van der Veldt studied this labeled drug in lung patients (100). They studied the biodistribution of the drug and the tumor uptake. It is interesting to note that some patients were treated before ([(11)^C^] docetaxel) with dexamethasone. A difference between the two groups in drug uptake has been recorded in favor of the patients pretreated with dexamethasone (100).

## Strategies for Modulating Interstitial Fluid Pressure and Other Factors That Obstacle Cancer Therapy

From these considerations, we can deduce that several factors may limit the delivery of the drugs to the tumor (see Figure 1) (Box 2). The first one is the increased distance that a drug must cross due to the volume increase. The second is the interstitial pressure (TIFP) that apparently produces a current flow from inside to outside of the tumor mass (60). This flow from the inside toward the outside slows the transportation of the drug from inside the vessel toward the tumor mass. TIFP is not only an obstacle to conventional therapy (chemotherapy) (101) but also hinders monoclonal antibodies (102, 103), nanoparticles (104, 105), and radionuclides delivery (106).

The pressure exerted by TIF is the result of two components the liquid component (*gel phase*) and the *solid phase* (cancer cells plus ECM) (107). Similar to liquid phase, the pressure of the solid phase is greater within the tumor mass than outside. Solid phase can collapse the blood vessel and is responsible for the lack of nourishment (and drugs) in particular areas of the tumor mass. Before listing and showing the studies conducted to decrease these two components (see Table 3), it is necessary to mention two studies that clearly illustrate that the modification of the interstitial pressure is indeed associated with a therapeutic improvement. The experimental study was conducted by Salnikov et al. (108). The authors examined two experimental tumors: syngenic rat colonic carcinoma (PROb) and dimethyl-benza-antthracene-induced rat mammary carcinoma (DMBA) treated with prostaglandin of E1 type (PGE1) and radiolabeled 5-FU [3H]5-FU. The authors clearly demonstrated that PGE1 exerted a substantial reduction of TIFP increasing the delivery of 5-FU, and the effect was not dependent on immune response or changes in tumor vascularity. The other study was conducted by Curti et al. (95) on nodules of patients with melanoma and lymphoma. Interstitial pressure was measured using the wick-in-needle technique. The sizes of the nodules were studied with ultrasound (US), and tumors were followed over time. Tumors were treated with either chemotherapy regimens rather than with immunotherapy. Responsiveness was correlated with IFP. In Table 3, all the methods (drugs, natural substances, and physical methods of treatment) used to modify TIF are illustrated. We have, also tried to show the human and animal studies, and to distinguish between the IFP exerted by the gel and solid phase and the possible clinical relevance.

**Table 3:** **Drugs, physical methods of cure, and natural drugs used to decrease tumor interstitial fluid pressure (TIFP), IFF, and VP**.

Drugs	IFP gel phase	IFP solid phase	Human studies	Animal studies	Effects	Reference	CR
**Angiogenesis inhibitors**							
Bezacizumab	↓			ѵ	VN	Ariffin	+
Sorafenib	↓			ѵ	VN	Ariffin	+
Imatimib	↓			ѵ		Ariffin	+
Block of receptor-2	↓			ѵ	VN↑Nano ≤12 nm	Chauhan	+
**Vasoactive agents**							
Hidralazine	↓			ѵ	↓ IFP not correlated to tumor volume	Podobnik	
Hidralazine	↓			ѵ	↑ Oxygenation	Jarm	
**Vascular disrupting agents**							
ZD6126						Skliarenko	
Combretastatin-A4	↓			ѵ		Ley	
**Chemotherapy**							
Chemo immunotherapy	↓		Melanoma lymphoma		↓ Responders	Curti	
Taxanes	↓			ѵ	↓	Bronstad	+
Taxanes	↓	↓		ѵ	↓	Griffon-Etiennie	+
Paclitaxel	↓		Breast cancer		↓	Taghian	+
PGE1	↓			ѵ	↓	Salnikov	
Dexamethasone	↓			ѵ	↓	Kristjansen	++
**Physical methods**							
Hyperthermia	↓			ѵ		Leunig	++
Hyperthermia				ѵ	↑ Oxygenation	Sen	++
Hyperthermia				ѵ	↑ MOABs	Jain M	++
Hyperthermia				ѵ	↑ Extravasation nanoparticles	Kong	++
Radiotherapy	↓			ѵ	↓ IFP correlated to radiocurability	Rofstad	++
US	↓			ѵ	↑ Gene therapy	Ziadloo Yuh	++
PDT	↓			ѵ	↑ Delivery of liposomial doxorubicin	Perentes	++
PDT	↓			ѵ	↓ IFP time dependent	Leunig et al. (109)	++
**Drugs acting on cellular matrix and cell density (CD)**							
Collegenases		↓		ѵ	↑ MOABs	Eikenes	
Hyaluronidase		↓		ѵ	↑ Liposomal doxorubicin	Eikenes	
Hyaluronidase				↓ CD	↑	Croix	
Losartan		↓		ѵ		Diop- Frimpong	++
TGF-β inhibitors		↓		ѵ	↑ Of chemotherapy/nano drugs delivery	Papageorgis	+
FAP vaccine		↓			↑ 70% drug uptake	Loffler	
**Natural substances**							
EGCG	↓			ѵ	↑ Activity cisplatin	Deng	++
↑ Oxygenation
w-3 FAs	↓			ѵ	↑ Activity of docetaxel	Kornfeld	++
↓ Activity of vascular NOS
**Drugs or physical methods acting on IFF**							
HT	↑ C			ѵ	↑ Nanoparticles extravasation	Kong, Leunig	++
CED	↑ C				↑ Convection (bypass of BB)	Saito,	+
CED	↑ C		ѵ			Vandergrift	+
US	↑ Convection					Frenkel	++
Angiotensin inhibitors	↓ FF	↓				Chauhan et al. (110)	++
VEGFR-3	↓ FF				↓ Lymphangiogenesis	Tammela	+
**Drugs acting on vascular permeability (VP)**							
Angiopoietin	↓					Gavard	
Bezacizumab	↓					Gerstner, Pishko	
Notch ligand Delta-like4	↓					Li; Azzi	

*BB, blood brain barrier; IFP, interstitial fluid pressure; IFF, interstitial fluid flow; EGCG, epigallocathechin-3-gallate; W-3FAs, omega-3 fatty acids; VN, vascular normalization; CL, capillary leakage; ↓, decrease; ↑, increase; ↑ C, increased convection; CD, cell density; CED, convection enhanced delivery; CR, clinical relevance, + with certain side effects, ++ with scarce side effects; NOS, nitric oxide synthase; MOABs, monoclonal antibodies*.

### The concept of tumor vascular normalization

Jain (111) introduced the concept of normalization of tumor vasculature. Jain and colleagues noted that using angiogenesis inhibitors an increase in oxygenation and drug delivery associated with a decrease in interstitial pressure. They also noted that an improvement in the release of nanoparticles with diameters ≤12 nm, disfavoring those with diameters >125 nm (112). The authors point out, however, that the effect of normalization is dose and time dependent (113). In fact, studies that employed high doses of bevacizumab showed that not only the tumor growth was slowed but also the coverage of vessels by pericytes was increased, thus decreasing the arrival of monoclonal antibodies to the tumor (114, 115). Huang (113) also emphasized the possibility of using the inhibition of angiogenesis as an immunomodulator as the abnormal vascularization that generates hypoxia also generates an immunosuppressive and inflamed TME (116). In addition to increased drug efficacy, Shrimali et al. (117) showed in a mouse model of melanoma that inhibition of angiogenesis increases lymphocytic infiltration and the effectiveness of adoptive cell therapy. In addition to immunotherapy, some chemotherapeutics, such as carboplatin and paclitaxel, enhance their effectiveness in the presence of an anti-angiogenic therapy. Heist et al. showed that associating anti-VEGF with chemotherapy improved the survival and the free period of illness in patients with advanced non-small lung cancer. It was also shown that it is possible to follow these patients with appropriate marks. For example, a high value of placental-derived growth (PIG) factor is associated with treatment with VEGF, and a high value of the receptor 1 of VEGF (VEGFR1) before treatment with bevacizumab with chemotherapy is a negative prognostic index (118).

### Vascular disrupting agents

Vascular disrupting agent (VDA) is a new class of drugs that affect the preexisting vasculature in the tumor mass (119). They mainly act by disrupting the integrity of the endothelial cytoskeleton inducing apoptosis and necrosis. As outlined by Ariffin (57), necrosis is able to increase hydraulic conductivity by indirectly decreasing TIFP. Two studies seem particularly interesting that confirm the effect of VDA on the interstitial pressure. The first is an experimental study on two tumors transplanted in mice, murine fibrosarcoma tumors (KHT-C) and a line of human cervical cancer (CaSki). These two tumors were treated with a binding agent, tubulin ZD6126, and interstitial pressure was measured using the wick-in-needle method (120). The TIFP behaved differently in the two types of tumor. The KHT-C tumors showed a sudden drop in TIFP 1 h after treatment, with a gradual slope and lifts to pre-treatment values about 3 h later. A drop of 25% of the pre-treatment values occurred after 72 h. The TIFP in the CaSki tumors decreased gradually over time, instead, reaching a value of −30% after 72 h. The mice with the two types of transplanted tumors showed a significant increase in survival after treatment (120). The second study was conducted on C3H mammary tumors grown subcutaneously in the foot of female CDF1 mice. The mice were treated with a single intraperitoneal injection of CA4DP. Tumor perfusion was recorded using a Laser Doppler flowmetry, and TIFP was measured continuously using the wick-in-needle technique (121). TIFP decreased rapidly after the treatment, followed by a concomitant reduction in tumor perfusion. According to the authors, perfusion increase was not TIFP dependent.

### Vasodilators

Several drugs with vasodilator activity have been shown in experimental studies to be able to decrease the interstitial pressure (122). In any case, a premise is necessary because the behavior of these drugs is not unique. Tumor vessels are devoid of innervation and part of tumor vasculature is that of the host, that is innervated and responsive to pharmacological stimuli. As pointed out by Vaupel (123), the effect produced by vasodilators depends on the positioning of the vessels of the host with those neo-formed. In fact, if the circulation of the tumor and that of the host are in parallel, the effect of the vasoactive drug is a decrease of flow in tumor; if the movement is in series, the vasoactive drugs produce an increase of flow into the tumor mass (123). Studies by Podobnik on SAF and LPB tumors have reported a decrease of TIFP in the presence of hydralazine. The decrease in interstitial pressure was not correlated with tumor volume (124). Studies by Jarm et al. have confirmed the positive association between hydralazine and TIFP but have also reported a decrease in tumor oxygenation (125). This last effect is probably due to steal phenomena and to the observations made by Vaupel (123).

### Chemotherapy

The low-molecular weight drugs used in chemotherapy reach the tumor by diffusion through the capillaries toward the tumor mass. Drugs with a higher molecular weight (i.e., monoclonal antibodies, nanoparticles, immune system cells) reach the tumor mass by convention (see Table 2). The most studied class of chemotherapeutics was the taxanes (paclitaxel and docetaxel) (126, 127). The Bronstad group has studied how taxane influence the ECM composition, specifically B1 integrins. They noted that the inhibitory effect on TIFP is linked to the action on the integrin, that both paclitaxel and docetaxel decrease TIFP in a dose-dependent manner, and that the fixing of actin filaments by phalloidin abolishes the effect of paclitaxel. The group of Griffon–Etienne has studied the effects of the taxanes on TIFP of two experimental tumors: the murine mammary carcinoma (MCa-IV) and the human sarcoma HSTS-26T. They determined that the taxane was able to decrease both the cell density (solid phase) and the gel phase. Taghian et al. studied the effects of paclitaxel on patients known to suffer from breast cancer (128). These authors noted that the decrease in interstitial pressure was followed by an improvement in oxygenation and that the effect was independent of tumor volume. Furthermore, the authors noted that only the paclitaxel seemed to achieve this effect. In fact, the concomitant administration of doxorubicin did not show any effect on TIFP and oxygenation.

Dexamethasone is a synthetic glucocorticoid used before or concomitantly with chemotherapy to reduce side effects (129) or increase the antitumor activity of certain drugs, such as carboplatin and gemcitabine (130). In addition to these activities, dexamethasone has been shown to decrease interstitial pressure. This activity study was conducted in SCID mice transplanted with tumor line LS174T. Two weeks after transplantation, mice were treated daily with intraperitoneal dexamethasone. A significant reduction in TIFP due to a reduced microvascular permeability and vascular hydraulic conductivity was obtained compared to a control group (131).

### Physical methods

#### Hyperthermia

Hyperthermia (HT) is a method of treating tumors using heat (132). Leunig et al. treated with HT several Amelanotic melanoma (A-Mel-3) implanted into the dorsal skin of Syrian golden hamsters. They studied the effect of HT on TIFP by using the wick-in-needle technique (133). They noted that the reduction of TIFP was temperature and time dependent, and the biological response was correlated with the TIFP reduction. Recently, Sen et al. (134) demonstrated similar results in several murine models. In addition, the authors noted that the reduction of TIFP was associated with an increase in perfusion and a sustained reduction of hypoxia. This reduction of hypoxia has led to a considerable improvement when radiotherapy was administered 24 h after HT.

#### Radiotherapy

Radiation therapy with chemotherapy is the standard therapies commonly used to treat solid tumors. Human melanoma xenografts transplanted intradermally or in window chamber preparations in BALB/c nu/nu mice were studied and treated with radiotherapy according their TIFP value. Mice with higher TIFP were treated with a higher radiation dose compared to those with a lower IFP. This indicates that a strong relationship exists between TIFP and radiocurability (135). The work of Rofstad et al. has associated the radiocurability to the value of the interstitial pressure, not taking into account that the radiotherapy is able to decrease the TIFP. Znati et al. (136) have highlighted this fact, and showed in xenografts of LS174T human colon adenocarcinoma grown in the right flank of nude (BALB/c) mice, that TIFP and tumor oxygenation were lowered by radiotherapy (RT). This effect was dose dependent, and it was necessary to give a dose between 10 and 15 Gy at minimum. The TIFP decrease and oxygen increase were dose dependent but not volume dependent. Anyway, Multhoff and Vaupel have doubts about the consistency of the data when applied to humans. They argue that the effects of radiation therapy on microcirculation as that on TIFP and drug delivery need more experimental data (75).

#### Photodynamic Therapy

Photodynamic therapy (PDT) is a minimally invasive method that uses a photosensitizer, the visible light of appropriate length and oxygen to generate oxygen free radicals (ROS). The ROS generated determine tumor cell death by apoptosis, disrupt the tumor vasculature, and generate a local inflammation and antitumor immunity (137, 138). Another positive effect of PDT is the capacity to lower TIFP. The first observations of this phenomenon were made by Leunig et al. in 1994 (109). These authors studied the time course of TIFP in nodules of melanoma implanted subcutaneously in four different districts of Syrian golden hamsters. They noted that the TIFP behaved differently depending on the time in which it was measured after the application of PDT. In fact, the TIFP exhibited an increase from 40 to 60% in the first 6 h, probably due to the impairment implemented on tumor microcirculation. After <24 h, the TIFP subsided by 50% compared to the control. A more recent study conducted on metastases of sarcoma in rats with subpleural implantations showed attractive valuations in Fisher rats (139). The PDT was able to decrease the TIFP in tumor nodules but not in the lung, and this was associated with a greater distribution of epirubicin in the tumor mass. In summary, the decrease of TIFP was associated with an increased convection of the drugs compared to controls.

#### Ultrasound Therapy

The US technique for depositing medication in the interstitial space is an emerging and promising method. In summary, drugs are activated via US and then released accurately and with decreased toxicity by using an imaging guide (140). Watson’s group (141) intended to study a method for reducing epithelial–mesenchymal transition (EMT), a situation with a propensity to metastasize and develop resistance to chemotherapy (142). In fact, these cells lose their polarity and adhesion capacity acquiring the capacity to migrate (143). In order to investigate the differences between epithelial tumors and tumors with EMT characteristics, liposomes containing radiolabeled 64 Cu can be used.

This allows the labeled liposomes to be followed by PET. Liposomes were found to accumulate in greater amounts (1.5-fold increase) in epithelial tumors compared to tumors with EMT characteristics without US application, whereas following US application; liposomes accumulated more in EMT compared to epithelial tumors. According to the authors (141), the nanoparticle accumulation was the result of TIFP reduction and of the increase in vascular permeability.

### Drugs acting on cellular matrix

The arrival of new pharmaceutical formulations, such as liposomes or nanoparticles with a diameter >10 nm, has highlighted the contribution of the composition of tumor matrix to drug delivery (144, 145). The deposition of the ECM is a complex process that involves two main components: cellular components (fibroblasts and inflammatory cells) and non-cellular components (146). In the presence of tumor cells, fibroblasts acquire special features to become the cancer-associated fibroblasts (CAFs). The stimulated CAFs produce the components of the matrix (147).

The main groups of components are glycoproteins, proteoglycans, and collagen. Each component has its physiological function, for example, seizure of growth factors, or provision of substrates to allow certain biochemical reactions or tissue differentiation (146, 148–150). A dynamic interaction exists between cancer cells, CAFs, and ECM, and their interaction is regulated by important cytokines, such as transforming growth factor-β (TGF-β) and PDGF (146). TGF-β stimulates CAFs to produce collagen type I, rendering the ECM stiffer and participating with VEGF to sustain angiogenesis and lymphangiogenesis (146).

The journey that a drug in the blood must take to reach the cancer cells in a body is similar to traveling through an obstacle course. Figure 1 shows all the obstacles that need to be overcome. In addition, the liquid part of the TIF drug should exceed cell densification and the composition of the ECM. Studies conducted by Netti have clearly demonstrated that the total content of collagen type I is more important than the content of glycosaminoglycans in halting the travel of the drugs (145). The author argues that less dense and less organized cellular matrix is capable of allowing a greater uptake of monoclonal and macromolecule cancer cells. Several authors aware of this factor tried to modulate the ECM. In order to obtain this, used enzymes were used to disrupt the ECM or the inhibitors of growth factors, such as PDGF and TGF- β, that control the formation of ECM (144, 151–155). Brekken et al. were among the first to demonstrate that injecting inside tumor enzymes, such as hyaluronidase or collagenase, which degrade the ECM was possible to modify the transvascular pressure (156). Eikenes demonstrated that collagenase (151) and hyaluronidase (152) were able to modulate the ECM and increase the monoclonal antibodies in the first case and lipoxomal doxorubicin in the second study. In both studies, the model studied was osteosarcoma xenografts, and the TIFP was measured by the wick-in-needle technique. Collagenase was injected peritoneally, whereas hyaluronidase was injected intratumorally and intravenously. The TIFP decrease behaved differently in the two groups. In the group treated with collagenase, the TIFP decreased as the microvascular pressure MVP, whereas in the group treated with hyaluronidase, the improved distribution of lipoxomal doxorubicin was induced by an increased transcapillary gradient. Diop-Frimpong et al. conducted an interesting work with an antihypertensive drug losartan (144). Losartan, an angiotensin II receptor antagonist, does not only exert antihypertensive activity but also has antifibrotic activity. This activity is carried out by reducing the activity of TGF-β1. The synthesis of collagen I decreases after 2 weeks and is dose dependent. As reported by Diop-Frimpong (144), collagen I synthesis was reduced under losartan in several experimental tumors, with minimal side effects. The results of experiments indicate that there is an amelioration of nanotherapeutics [(gene therapy) herpex simplex viruses] and pegylated liposomal doxorubicin uptake by tumor. Another molecule that is able to decrease TIFP is PDGF inhibitors. It seems that PDGF is able to increase IFP in the dermis by interfering with integrins and phosphatidylinositol-3* kinase signaling (157). Pietras et al. using A PDGF receptor tyrosine kinase inhibitor (STI571), increased the uptake of taxol in an experimental model of subcutaneous (s.c.) implanted KAT-4 tumors in SCID mice (154). Another group tested the same PDGF inhibitor (155) in a s.c. xenografts of human colorectal carcinoma in athymic mice treated with a radiolabeled antibody B72.3. They demonstrated an improvement of radioimmunotherapy uptake. The antibody uptake was more homogeneous and associated with an augmented radiosensitivity due to an increased oxygenation of the tumor mass. As previously described, TGF-β is one of the principal cytokines that regulate the ECM and collagen deposition. This last effect is obtained by converting fibroblasts into CAFs and stimulating them to produce lysyl oxidase, an enzyme that is able to stiffen collagen (153, 158). The authors suggest the use of inhibitors of TGF-β in tumors with strong desmoplasia because, in their view, it should result in increased perfusion. Emphasize, however, that the increase in perfusion would indirectly increase the capillary pressure (Pc, see Figure 1) with an increase of filtration. Increased filtration increases the interstitial fluid and then would form a vicious circle. Furthermore, the block of TGF-β was also shown to increase metastasis. A combined treatment with chemotherapeutic type taxanes, losartan, and inhibitors of TGF-β should optimize the result and not trigger the vicious circle (153). An example of this association is the work of Zhong (159). Furthermore, these authors using chemotherapeutic cyclophosphamide not only controlled the metastases but also enhanced antitumor immunity. Loeffler et al. have addressed the problem of the matrix and deposition of collagen type I, trying to regulate fibroblasts the fabricators of collagen. They used a DNA vaccine for the oral fibroblast activating protein (FAP), which is overexpressed in tumor stroma. With this method, they were able to decrease the production of collagen and increase by 70% the uptake of chemotherapy by the tumor cells (160). Bouzin et al. emphasize that a difference exist between collagenase and hyaluronidase (161). According to these authors, the degradation of fibrillar collagen is more efficient on drug uptake than the degradation of hyaluronan. Collagenase, as previous discussed, increases the uptake of monoclonal antibodies and the efficiency of herpes simplex virus (161).

### Natural substances (nutraceuticals)

The use of nutraceuticals as adjuvant to traditional cancer therapy is increasing. Two substances have been studied regarding the possibility to lower the TIFP, which are the epigallocatechin-3-gallate (EGCG) (162) and omega-3 fatty acids (163). EGCG is a molecule with an anti-angiogenic activity. In fact, EGCG has the capacity to regulate the receptor of VEGF and to modulate the angiopoietins 1 and 2 (Ang-1; Ang-2) (164). Ang-2 may loosen the endothelial cells’ junctions rendering more leaky tumor vessels. EGCG inhibited both Ang-1 and Ang-2 but had a greater effect on Ang-2, thus, decreasing the tumor vessel’s permeability and, in effect, TIFP level. Deng demonstrated this phenomenon in mice with xenografts in A549 cells. TIFP was measured using the wick-in-needle technique, and hypoxia was measured using polarographic needle electrodes. EGCG showed a synergism with cisplatin, indicating the possibilities of using EGCG to decrease TIFP and as a sensitizer to chemotherapy (162). Kornfeld used omega-3 fatty acids (w-3 FAs) to control TIFP. As known from the literature, omega-3 fatty acids (w-3) have multiple effects on cancer prevention and therapy (165). Among the most important effects were anti-angiogenic activity and the modification of nitric oxide synthase. Nitric oxide (NO) and VEGF are responsible for vascular hyperpermeability (163, 165). The inhibition or modulation of these two factors normalizes the abnormal vascular structure of tumors, as pointed out by Jain. Based on this, Kornfeld (163) studied a breast cancer model in mice. Kornfeld et al. subjected a group to a diet rich in w-3 polyunsaturated fatty acids (50% DHA 20% EPA), another group with this diet and docetaxel, and another group as the control. They then studied the synthesis of NO *in vitro* using a human umbilical vein. The group treated with w-3 + docetaxel showed vasculature normalization, decreased drug resistance, improved drug delivery, and a change in nitric oxide activity.

### Cell packing, density

At the end, a drug must overcome another obstacle, the difficulty of penetrating and accumulating at the necessary concentration in the tumor cell mass. The difficulty level is higher for protein binding drugs, such as doxorubicin and paclitaxel (166). The factor that determines decreased penetration is cell density (167). The authors conclude that drug penetration in a 3D structure is 5- to 10-fold less effective than in a monolayer. Tredan et al. outline further this concept and consider cell density one of the causes of tumor drug resistance associated with TME characteristics (i.e., hypoxia, extracellular acidity) (168). We can define TME (cell density) as a set of cancer cells, normal cells, tumor/normal vascular structure, and ECM dense packed together. Furthermore, tumor cells can be distinguished between well oxygenated, hypoxic, and moderately oxygenated embedded in an ECM rich in cytokines and growth factors. As previously mentioned, ECM is denser than normal matrix and related to the deposition of collagen (145). This dense complex of tumor cells, normal cells, and ECM forms a special microenvironment that hinders further penetration of drugs, resulting in a situation of drug resistance (150, 168–171). These authors point out that the methods to study the tumor drugs must be ameliorated. Culture studies on monolayers should be replaced with studies with spheroids or with cultures on multilayers and *in vivo* methods (171). An example of these methodologies is the recent work of Grantab et al. (172). These authors used two variants of HCT-8 human colon carcinoma xenografted in nude mice and a multilayered cell culture. One of these variants had a lower packing density, and the other had greater density. They treated the two groups with bortezomid and showed improvement in drug penetration, an increased cytotoxicity, and a reduction in cell density. A decrease in TIFP was also noted. Another study by Croix demonstrated that hyaluronidase was able to reverse the cell density, increasing tumor cell sensitivity to various chemotherapeutics (173).

### Drugs acting on IFF

#### Drugs that Augment Convection

##### Convection enhanced delivery

With convection enhanced delivery (CED) we intend a treatment able to overcome the IFF current from the tumor toward the environment or the lymphatic vessels. This kind of therapy, normally, is used to treat glioblastoma overcoming the obstacle of the blood brain barrier (BBB) (174). The method consists of the insertion into the brain tissue of a small caliber catheter stereotactically guided toward the tumor mass. The first to describe this methodology was Bobo et al. (175) in late 1994. Debinsky et al. described the different kinds of catheters used for CED (176). Saito et al. (177) developed special liposomes labeled with gadolinium and a fluorescent indicator. They followed and monitored their distribution inside the brain tumors of rats after a microinfusion under pressure. The technique (CED) increased the local drug delivery and demonstrated its clinical use. The molecules (radioisotope labeled drugs, immunotoxins) were pumped through this catheter to penetrate the brain parenchyma. Vandergrift et al. (178) report the phase I, II, and III clinical trials conducted with CED technique. Phases I and II have been completed and have shown promising results consisting of partial response while avoiding side effects. Limitations of the techniques are linked to the tumor’s location and are justified by the tumor’s aggressiveness. The catheters generally must provide sufficient infusate and prevent reflux. The leakage of refluxed infusate is the most important side effects associated to the possibility of infections (179).

Other two methods can augment convection: HT and US. As previously reported, HT increases drugs’ extravasation and accumulation into the tumor mass, also decreasing TIFP (133, 180). Another physical method that is able to increase convection is the use of US (181).

#### Drugs that Decrease IFF

Another way to increase the arrival of drugs to the tumor is to hinder or decrease the effect of the IFF by increasing the pressure in the vessels that supply the tumors. This result is achieved using antagonists against the angiotensin II receptors (110). Experimental studies have demonstrated that these antagonists can increase the perfusion of the tumor vessels by decreasing the IFF indirectly. Other positive effects are an enhancement of oxygen delivery to the tumor and a potentiation of chemotherapy, such as 5-FU in AK4.4 pancreatic tumors. The increased release of drugs has also been shown in pancreatic tumors, human breast, and skin cancers. What these tumors have in common is the high concentration of collagen in the ECM that the angiotensin inhibitors can reduce. As to chemotherapy, the authors demonstrated that the distribution of the nanoparticles, liposomal doxorubicin, and oncolytic virus also undergoes an improvement (144). Another way to decrease the efflux of IFF is to inhibit the transport by the lymphatic system. An example of this approach is the use of monoclonal antibodies that block VEGF receptor type 3 (182, 183). As demonstrated by Alitalo et al. (182), VEGF receptor type 3 is an essential signaling mechanism in lymphangiogenesis and tumor progression. The decreased drainage via the lymphatic system reduces the IFF and drug removal (65).

### Drugs acting on vascular permeability

The mechanisms that regulate the vascular endothelial permeability are essentially two: the tight junctions (TJ) and the adherent junctions (AJ). The TJ represent the barrier capable of regulating cell migration, while AJ maintain the physical junction between the endothelial cells. Among the various trans membrane proteins belonging to AJ the most important are the VE-cadherin proteins and anchor proteins α, β, υ, and p-120 catenins. The VE-cadherin bind to β-catenins and β to α-catenins forming a complex that interact with the proteins of the cytoskeleton. This complex interacts with regulatory proteins, such as Src kinase and several phosphatases that modulate the AJ junctions (184, 185). The restoration of vascular permeability is here briefly described as we invite the readers to see the review of Azzi et al. (184) for a more complete discussion of the argument.

#### Angiopoietin

Vascular endothelial growth factor is the principal enhancer of the endothelial permeability. It produces this effect acting on VE-cadherin through a mechanism Src dependent. The angiopoietin blocks the destabilization of VE-cadherin although collaborates on angiogenesis. The angiopoietin in conclusion has angiogenic activity as VEGF but otherwise has no activity on vascular permeability (186).

#### Bevacizumab

Vascular normalization as outlined by Jain (187) is a method able to carry for limited period to normality the abnormal tumor vasculature. For limited period, we intend the period during which anti-angiogenetic drugs exert their effect. Jain and his group demonstrated that normalization in glioblastoma is able to decrease hypoxia, to increase drug arrival and survival. Survival was associated to an increase in tumor perfusion (188). Gerstner and Batchelor reported similar results and outline that brain edema, which is the result of BBB disruption, was less using one of most known anti-angiogenetic monoclonals bezacizumab (189). Pishko et al. (190) using bezacizumab demonstrated vascular normalization in rat model of human cancer brain metastases.

#### Notch Ligand Delta-Like 4

Li et al. in the late 2007 (191) founded that beyond VEGF Notch ligand Delta-like 4 (DL4) plays a role in angiogenesis. DL4 is a negative regulator of angiogenesis reducing the quantity of neovessels. DL4 improves vascular function but does not interfere with the activity of bezacizumab and reduce both angiogenesis and the vascular effect (permeability) of VEGF (184).

## Conclusion

The status of the TME should be of primary importance for oncologists (170). The possibility of studying it *in vivo* by MRI may contribute to their ability to choose the most appropriate drugs use (chemotherapy/natural drugs or monoclonal antibodies). These drugs are able to modify TME and, consequently, the tumor interstitium (192–197). In fact, MRI offers the possibility of knowing the density of the matrix, the forces that govern the distribution of drugs, the perfusion, the pH of the microenvironment, and its metabolic status (195, 196, 198). As with conformal radiotherapy (CFRT), a computerized, imaging reconstruction platform can be used and analyzed by a staff composed of physicists, biologists, and physicians. In the near future, it will be possible to comprehend the TME of any patient and apply a personalized treatment (196). As to the CFRT, it is clear that semi-automatic image analysis methods will provide a better understanding of angiogenesis and modulation of interstitial pressure (98, 199). There is no doubt that mathematical and silicon models will be helpful in this process, transitioning experimental studies from the laboratory to the clinic (82, 200). As reported by Kuszyk (199), the barriers that obstruct drug delivery: (a) the vascular transport, (b) the crossing of vessel walls, and (c) the distribution inside the interstitium can be studied by MRI.

We have considered, in this review, drugs that can interfere with interstitial pressure. In Table 3, we have introduced the concept of clinical relevance (+). By this, we mean drugs or methods currently used and able to achieve a higher deposition of drugs. Some methods that we stressed (++) seem more usable, with fewer side effects. It is interesting to note that some nutraceuticals have activities on TIFP, which partially explains their activities in sensitizing tumor cells to chemotherapy. Many new studies that take into account these nutraceuticals would be useful and desirable. However, we must avoid an incorrect association that would decrease some pharmacological activities. Another consideration is that cancer patients often have other associated disorders, such as diabetes or heart disease (201). In the case of diabetes, it would be interesting to see if some medications, such as metformin, can be used for reducing collagen synthesis, fibrosis (202), or angiogenesis (203). In this review, the dynamic process of angiogenesis has not described except in summary form. Neoangiogenesis is definitely the most important phenomenon in the genesis of the increased interstitial pressure and in tumor progression (61). In fact, the growth, irregular and imperfect of the new endothelium, is responsible for the increased permeability (27) and for the lack of oxygenation of some tumor areas and the perpetuation of the phenomenon: angiogenesis, hypoxia, and angiogenesis (204). The abnormal and unregulated growth of 3D tumors linked to irregular spraying leads to the inadequate distribution of medications and, consequently, a resistance to them. The opportunity to use more drugs (natural or synthetic) can lead to a normalization of tumor blood circulation and to important effects, such as an increased release of drugs and an increased immune response (187). The improvement of the imaging methods will permit to test clinically new therapies in a quicker time. This method will enable us shortly to use the best therapies for individuals (precision medicine) knowing the status of the patient’s TME.

An interesting and useful method that is an example of ongoing research between experimental measurements and possible future applications at the bedside is the study of Leguerney et al. (205). These authors performed the measurement of tumor volume, perfusion, and TIFP on 60 mice xenografted with B16F10. They treated the animals with an association of sorafenib and bezacizumab. The two drugs as reported by the authors to have a positive association with a variety of tumors. Perfusion and vasculature were measured by quantitative dynamic contrast-enhanced ultrasonography (DCE-US), whereas TIFP measured with a fiberoptic probes. The authors demonstrated that TIFP variations were predictive of vascular changes and that a single measure of TIFP was sufficient for characterizing the entire tumor mass. Authors refer that no correlation present between TIFP value and tumor perfusion. This method is interesting for the following reasons. DCE-US is relatively inexpensive and used at the patient bed permitting to follow the application of anti-angiogenic drugs. Disadvantages are probably the difficultness to measure the parameters in deep-seated tumors and the invasive way of TIFP measure. Another disadvantage is that the technique is not applicable to all the patients and probably not easy reproducible.

A phenomenon that we have not analyzed but correlated to the increased permeability of tumor vessel is the so-called retention enhancement effect (EPR) of Maeda. This phenomenon regards drugs with a molecular weight ≥12,000 Da, nanoparticles and liposomes (206). As outlined by Jang et al. EPR is a passive tumor targeting approach (207). The EPR effect is another example of interaction between TIFP and IFF. As discussed, TIFP is a uniformly centripetal pressure, whereas IFF has a centrifugal behavior. However, TIFP is sufficiently elevated compared to the pressure difference existing between the tumor vessels and the normal tissue around a tumor. This in a certain sense hampers the transport away of the molecules adjacent to the vessel area, keeping them in that area for a longer time (EPR effect) (82).

Vascular normalization seems to be the simplest method of treatment for controlling TIF formation and TIFP. However, Ribatti (208) has criticized vascular normalization. In fact, this author outlines that the vessel normalization’s restoration followed by normalization of vessel permeability may become an obstacle to the subsequent chemotherapy (208). This is true, but we think that a better comprehension of TIF formation, TME, and of the forces that govern their interactions, associated with vascular normalization and the new imaging methods and computational models, may represent the future of cancer therapy. Another way to lowering TIFP and ameliorating convective flux is the use of collagenase. The work of Gade et al. (99) is the one of the best example to transport into clinics. In fact, the authors followed with MRI the increase in delivery of 5-FU to HT29 human colorectal tumors grown s.c. in mice after administration of collagenase. They demonstrated a 50% increase in 5-FU increase in mice treated with collagenase compared to a control (99). HT clinically if properly used is free of serious side effects. HT associated with chemotherapy and radiotherapy increases their antitumoral effects. We refer the reader to Datta et al. (209) and Hurwitz et al. (210) to understand the mechanisms that determine these positive associations. We can point out that the right combination of them (i.e., cisplatinum–taxanes–HT–radiotherapy) can only lead to a better therapeutic index. In fact, if we look at all these various factors individually they lower the interstitial pressure augmenting drug delivery to the tumor. The experimental group directed by Repasky (134) comforts us. They are indicating that an appropriate use of oncology therapies and imaging methods already in use can cause us to take a big leap forward in the clinic.

## Conflict of Interest Statement

The authors declare that the research was conducted in the absence of any commercial or financial relationships that could be construed as a potential conflict of interest.
